# Comparative Study of Ropivacaine and Ropivacaine With Dexmedetomidine in Transversus Abdominis Plane (TAP) Block for Post-operative Analgesia in Patients Undergoing Cesarean Sections

**DOI:** 10.7759/cureus.65588

**Published:** 2024-07-28

**Authors:** Deb Sanjay Nag, Priti Gehlot, Prashant Sharma, Himanshu Kumar, Umesh Kumar Singh

**Affiliations:** 1 Anesthesiology, Tata Main Hospital, Jamshedpur, IND; 2 Anesthesiology, Steel City Clinic and Research Center, Jamshedpur, IND; 3 Anesthesiology, Manipal Tata Medical College, Jamshedpur, IND

**Keywords:** postoperative pain, cesarean section (cs), bupivacaine ropivacaine, dexmedetomidine, transversus abdominis plane block (tap block)

## Abstract

Background

Adequate post-operative analgesia in the obstetric patient is necessary to facilitate breastfeeding and the care of the newborn. Considering the limitations of intravenous analgesic options such as non-steroidal anti-inflammatory drugs (NSAIDs) and opioids, other alternatives have been tried for offering better analgesia with fewer potential side effects. Transversus abdominis plane (TAP) block is one such option that has been tried with various local anesthetic drugs, either alone or in combination with other adjuvants. The addition of dexmedetomidine to bupivacaine in TAP block has been shown to prolong the duration of post-operative analgesia when compared to local anesthetic alone. This study was conducted to determine the efficacy of dexmedetomidine, as an adjuvant to ropivacaine, when administered in TAP block in patients undergoing cesarean section.

Methodology

The study was a prospective, randomized, parallel assignment, triple-blinded controlled trial. Hundred patients posted for elective lower segment cesarean section, fulfilling the inclusion criteria, were randomly divided into two equal groups, group R and group RD, comprising 50 patients each. Patients in group R were administered bilateral TAP block by landmark technique using ropivacaine alone, whereas patients in group RD were administered TAP block with dexmedetomidine 1 micrograms/kg, in addition to a similar dose of ropivacaine. Mean arterial pressure (MAP), heart rate (HR), visual analog scale (VAS)-R (pain score on VAS scale at rest), VAS-C (pain score on VAS scale on coughing), nausea and vomiting, and Ramsay sedation score were recorded on admission to post-operative care unit (PACU), and at first, fourth, eighth, 12th, 18th, and 24th hours post-operatively. Rescue analgesia was provided with intravenous morphine. Short Assessment of Patient Satisfaction Score (SAPS) was noted on a five-point scale after 24 hours based on patient satisfaction regarding the quality of post-operative analgesia.

Results

While there was no significant difference between groups R and RD with respect to VAS-C and VAS-R immediately after shifting and at the first, fourth, and eighth hours, a significant difference was observed at the 12th and 18th hours post-operatively. After 24 hours, no significant difference was observed between groups R and RD with respect to VAS-C and VAS-R. While 50% of patients needed rescue analgesia in group R, only 28% of patients needed rescue analgesia in group RD. There was significantly better patient satisfaction measured by the Short Assessment of Patient Satisfaction Score (SAPS) with respect to the quality of analgesia in patients in group RD as compared to those in group R.

Conclusions

The addition of dexmedetomidine to ropivacaine increased the duration of post-operative analgesia up to 18 hours post-operatively in cases of elective lower segment cesarean section. Also, the quality of post-operative analgesia is better in such patients, as shown by a significant difference in patient satisfaction scores between the two groups.

## Introduction

The ideal post-cesarean analgesic regime should be efficacious without impacting the ability of the mother to take care of the neonate and with minimal drug transfer through breast milk [1]. The addition of Paracetamol and non-steroidal anti-inflammatory drugs (NSAIDs) potentiates the effects of opioids, decreases its consumption, and reduces the side effects when systemic and neuraxial opioids are administered for post-cesarean delivery analgesia. The anti-inflammatory and antipyretic properties of NSAIDs may reduce visceral pain originating from the uterus, complementing the somatic wound pain relief from the opioids. NSAIDs must be used with caution because of the potential problems with bleeding, platelet dysfunction, and renal insufficiency, although practice varies widely in this regard [2].

Considering the limitations of the analgesic options discussed above, other alternatives have been tried to offer better analgesia with lesser potential side effects. Transversus abdominis plane (TAP) block is one such option, which has been tried with various local analgesic drugs either alone or in combination with other adjuvants. TAP block was introduced by Rafi [3] in 2001. He described it as a block delivering local anesthetics in the transversus abdominis plane, using the anatomical landmarks (iliac crest) by first identifying the lumbar triangle of Petit. Direct visualization of all anatomical structures, the needle, and the spread of local anesthetic by ultrasonographic guidance may be associated with an increased margin of safety and optimal block qualities [4]. However, the ready availability of ultrasound (USG) for this purpose might not be possible, specifically in developing countries [5]. Certain authors have quoted a better result with the “landmark technique,” resulting in a lesser narcotic requirement, and a Cochrane review published in 2010 [6] also failed to claim the superiority of USG over the “landmark technique.” Although ultrasound-guided TAP blocks are recommended these days, ultrasound machines are always not available in all operation theaters. The “landmark technique” can be performed even in low-resource settings when ultrasound machines are not available.

Both bupivacaine and ropivacaine have been used as the local anesthetic for performing TAP block in patients undergoing cesarean section [7]. Ropivacaine, however, is a safer drug than bupivacaine while retaining a similar quality and duration of sensory blockade. Various adjuvants had been used in previous studies, such as opioids (fentanyl, buprenorphine, morphine), vasoactive agents (epinephrine), alpha-2 agonists (clonidine, dexmedetomidine), non-steroidal anti-inflammatory drugs (parecoxib, ketorolac), dexamethasone and tramadol for prolongation of the analgesia offered by nerve block. Dexmedetomidine is a selective alpha-2 adrenergic agonist with both analgesic and sedative properties. It also has a vasoconstricting effect, thereby decreasing the systemic absorption of local anesthetics, and offers an anti-inflammatory effect. The addition of dexmedetomidine to bupivacaine in TAP block has also been shown to prolong post-operative analgesia with better pain control than the local anesthetic alone [8]. The chief objective of our study was to find out if the addition of dexmedetomidine to ropivacaine in TAP block prolonged the duration of post-operative analgesia and provided better pain control than the local anesthetic alone.

## Materials and methods

The study was a prospective, randomized, parallel assignment, triple-blinded controlled trial conducted over a period of six months from May 2015 to November 2015 in the Department of Obstetrics and Gynecology at Tata Main Hospital, Jamshedpur. The study was approved by the Institutional Ethics Committee with a unique ID of SR9851450, based on which it was registered on ClinicalTrials.gov, the registry of clinical trials run by the United States National Library of Medicine (NLM) at the National Institutes of Health, with an ID of NCT02472522. Written informed consent was obtained from those willing to participate in the study. A total of 100 patients with term pregnancy, aged above 18 years, belonging to the American Society of Anesthesiologists (ASA) Physical Status Classification System grades I-II, scheduled for elective lower segment cesarean sections, were included in the study. Randomization was done by computer-generated random numbers, and allocation concealment was done by sealed opaque envelopes. The study was triple-blinded, wherein the participant, care provider, and outcome assessor were unaware of the allocated study group. Patients who refused to participate in the study, those having known allergies to medications used in the study, patients having localized infection at the site of TAP block, patients with body mass index (BMI) > 35, patients having coagulopathies with contraindications to regional anesthesia, patients with a history of cardiac, respiratory, renal, hepatic failure, and patients with psychological disorders, were excluded from the study.

Hundred patients fulfilling the inclusion criteria, scheduled for elective lower segment cesarean section, were randomly divided into two groups, group R and group RD, comprising 50 patients each. All patients were administered subarachnoid block in the sitting position with bupivacaine heavy 0.5% (11 mg/2.2 ml) along with 25 micrograms of fentanyl. At the end of the surgery, a bilateral TAP block was administered using the landmark technique in the triangle of Petit. Excessive weight gain or obesity might obscure landmarks; therefore, we excluded patients with BMI >35 in our study.

The landmark technique, described by McDonnell et al., accesses the transversus abdominis plane via the lumbar triangle of Petit. This is a surface landmark bound by the external oblique muscle anteriorly, the latissimus dorsi muscle posteriorly, and the iliac crest inferiorly. The transversus abdominis plane was identified by a loss of resistance technique. The triangle of Petit was first identified as the area between the anterior border of the latissimus dorsi, the posterior border of the external oblique, and the iliac crest. The skin over this triangle was pierced with a blunt-tipped 2-inch 24 G needle until resistance was felt. This first resistance signified the needle tip entering the fascial extension of the external oblique muscle. Further advancement resulted in a loss of resistance, as the needle entered the fascial plane between the external and internal oblique muscle layers. Further advancement results in a second increased resistance as the needle traverses the fascial extension of the internal oblique. A second loss of resistance signified entry into the fascial plane of the transversus abdominis. After careful aspiration to exclude vascular puncture, the local anesthetic solution was injected. The TAP block was then performed on the opposite side using an identical technique. Patients in group R were administered a TAP block with 2.5 mg/kg of 0.75% ropivacaine diluted with 0.9% saline to a total volume of 40 ml (20 ml each side). Patients in group RD were administered a TAP block with 2.5 mg/kg of 0.75% ropivacaine with dexmedetomidine 1 micrograms/kg diluted with 0.9% saline to a total volume of 40 ml (20 ml each side).

Intra-operatively, baseline mean arterial pressure (MAP) and heart rate (HR) were recorded. MAP and HR at five, 10, 20, 30, 40, 50, and 60 minutes were also recorded. The duration of surgery was recorded in minutes (from the surgical incision to skin closure). 75 mg of intramuscular diclofenac sodium was administered immediately on shifting to the post-operative care unit (PACU), and a second dose was given 12 hours later. Morphine 6 mg was provided for rescue analgesia, with additional doses of 3 mg at 10-minute intervals till visual analog scale (VAS) was less than 3, or the development of adverse effects such as nausea, vomiting, respiratory depression (SpO_2_<92%, respiratory rate<10), or deep sedation (Ramsay sedation score more than 2). In the recovery area, the level of motor blockade was noted as per the modified Bromage Scale. The time from administration of the subarachnoid block to complete fading of the motor block (Bromage 0) was noted in minutes. The total duration of sensory loss due to subarachnoid block at the T10 level was assessed by a pin-prick test. The time duration after the TAP block, when rescue analgesia was first required, and the total morphine requirement in 24 hours post-operatively was noted. Any adverse effects of morphine, such as pruritus, nausea, and vomiting, were also noted. Ramsay score on a six-point scale was also observed.

Post-operative pain was noted on the visual analog scale (VAS). MAP, HR, VAS-R (VAS during rest), VAS-C (VAS on coughing), nausea and vomiting, and sedation score (RS) were recorded on admission to the post-anesthesia care unit (PACU), and at first, fourth, eighth, 12th, 18th, and 24th post-operative hours by an observer who was unaware of the study protocol. A Short Assessment of Patient Satisfaction Score (SAPS) was noted on a five-point scale at the end of 24 hours based on the feedback of the patient on post-operative analgesia. A SAPS score of 1 signified the patient being highly dissatisfied, whereas a score of 5 was assigned to patients who were highly satisfied. 

Statistical testing was conducted using the statistical package for the social science system version SPSS 17.0 (IBM Corp., Armonk, New York, USA). Continuous variables are presented as mean ± standard deviation (SD), and categorical variables are presented as absolute numbers and percentages. The comparison of normally distributed continuous variables between the groups was performed using the Student’s t-test. Nominal categorical data between the groups were compared using the Chi-squared test or Fisher’s exact test as appropriate. A 'p' value<0.05 was considered as statistically significant.

## Results

Demographically, there were no statistical differences between groups R and RD with respect to age, weight, height, and duration of surgery. Intra-operatively, there was no statistical difference in HR and MAP of patients in both groups. Also, the difference between groups R and RD with respect to the time to complete disappearance of motor block and duration of sensory loss at T10 level was not statistically significant. Post-operatively, there was no significant difference in MAP and HR between groups R and RD immediately after shifting, and at first, four, eight, 12, 18, and 24 hours post-operatively. 

**Figure 1:**
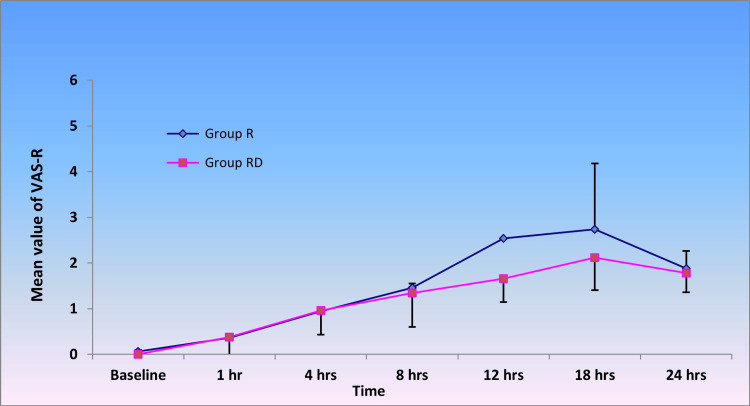
VAS-R on shifting and after first, fourth, eighth, 12th, 18th, and 24th hour post-operatively. A significant difference ('p' value<0.05) was observed between groups R and RD with respect to VAS-R at 12 hours (p<0.001) and 18 hours ('p' value=0.008) post-operatively. Student's t-test was used to obtain the 'p' value. VAS-R: Post-operative pain measured on the visual analog scale, during rest. Group R: Patients in this group were administered bilateral TAP block with ropivacaine. Group RD: Patients in this group were administered bilateral TAP block with ropivacaine along with dexmedetomidine.

**Figure 2:**
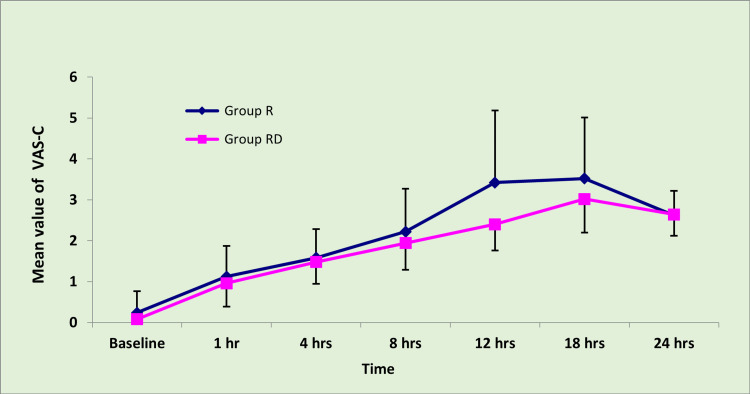
VAS-C on shifting and after first, fourth, eighth, 12th, 18th, and 24th hour post-operatively. A significant difference ('p' value<0.05) was observed between groups R and RD at 12 hours (p<0.001) and 18 hours ('p' value=0.008) post-operatively. Student's t-test was used to obtain the 'p' value. VAS-C: Post-operative pain measured on the visual analog scale, on coughing. Group R: Patients in this group were administered bilateral TAP block with ropivacaine. Group RD: Patients in this group were administered bilateral TAP block with ropivacaine along with dexmedetomidine.

There was no significant difference between groups R and RD with respect to VAS-R (Figure 1) and VAS-C (Figure 2) immediately after shifting and at first, four, and eight hours post-operatively. Nonetheless, a significant difference was observed at the 12th and 18th hours. With a ‘p’ value<0.05, this indicates that the addition of dexmedetomidine to ropivacaine can prolong the post-operative analgesic effect of TAP block up to 18 hours. Again, at the 24th hour, groups R and RD did not demonstrate any significant difference with respect to VAS-C and VAS-R. 

**Figure 3:**
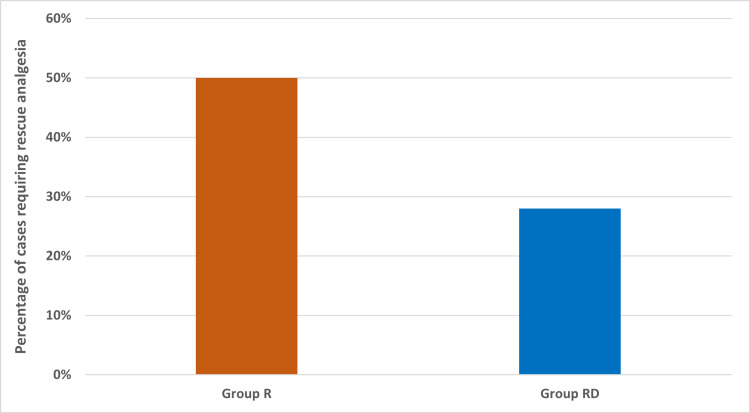
Requirement of rescue analgesia in groups R and RD. 50% of patients (25/50) needed rescue analgesia in group R, whereas only 28% of patients (14/50) needed rescue analgesia in group RD. There was a statistically significant difference between the two groups in the need for rescue analgesia ("p" value=0.024). Two proportion test was used to obtain the 'p' value. Group R: Patients in this group were administered bilateral TAP block with ropivacaine. Group RD: Patients in this group were administered bilateral TAP block with ropivacaine along with dexmedetomidine.

While 50% of patients (25/50) needed rescue analgesia in group R, only 28% of patients (14/50) needed rescue analgesia in group RD (Figure 3). As compared to group R, it was found that the addition of dexmedetomidine decreased the demand for rescue analgesia in group RD. There was a statistically significant difference between the two groups in the need for rescue analgesia ('p' value=0.024). Patients in group R needed rescue analgesia earlier, with a mean time to rescue analgesia in group R being 13.12 ± 3.53 hours, whereas in group RD, this was 18.21 ± 3.14 hours (p=0.001). Similarly, the mean of total morphine required in group R was 8.16 ± 2.94 mg and that required in group RD was 6.21 ± 0.80 mg (p=0.004). No major adverse effects were observed in the groups. Two patients had vomiting in group R, whereas only one patient had vomiting in group RD. One patient complained of nausea in group R, whereas no patient had any such complaint in group RD.

**Figure 4:**
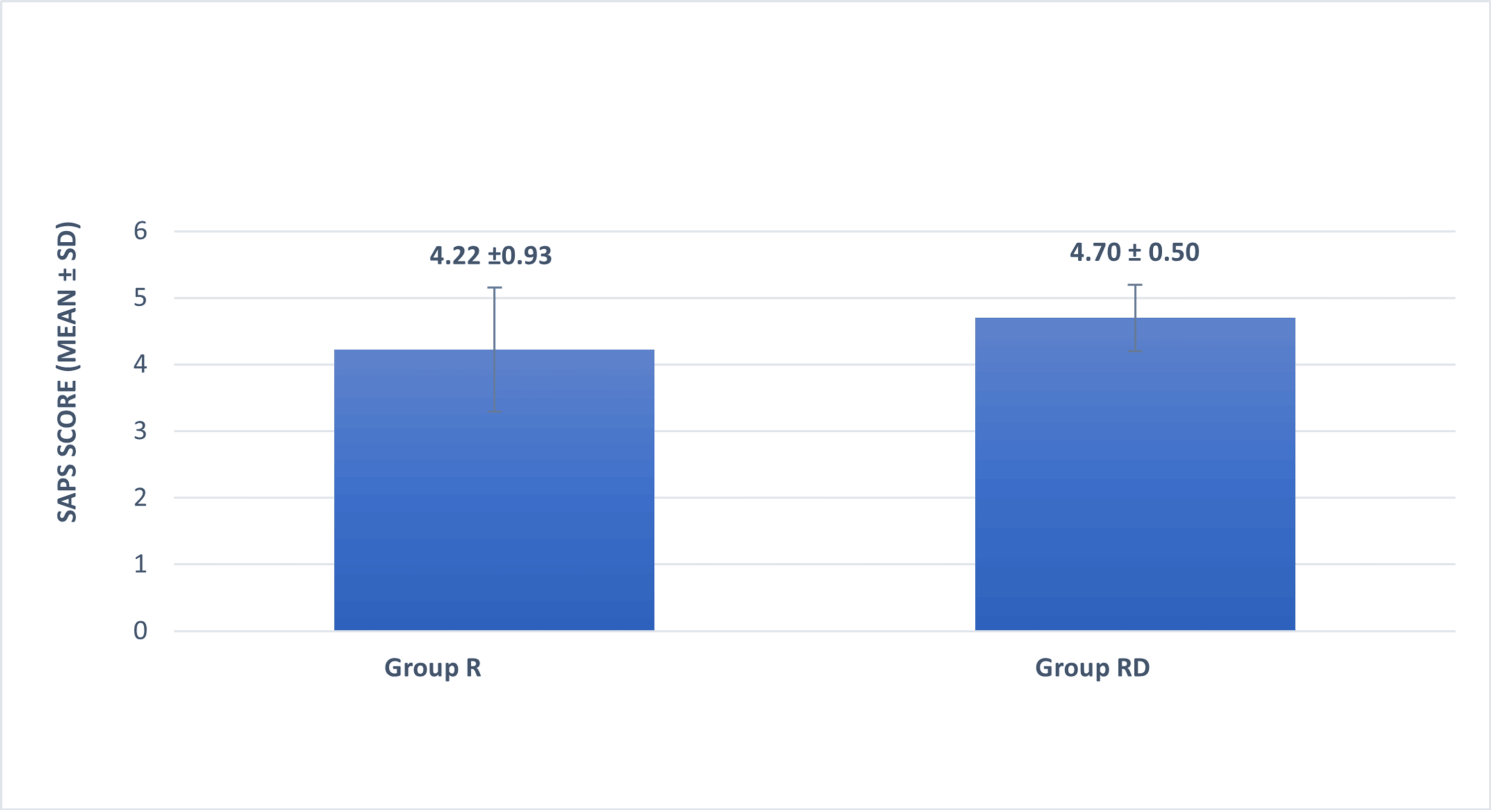
Comparison of the Short Assessment of Patient Satisfaction (SAPS) score between groups R and RD. Patients in group RD had a significantly better SAPS score ("p" value=0.002) as compared to patients in group R. Student's t-test was used to obtain the 'p' value. SAPS: Short Assessment of Patient Satisfaction Score. Group R: Patients in this group were administered bilateral TAP block with ropivacaine. Group RD: Patients in this group were administered bilateral TAP block with ropivacaine along with dexmedetomidine. SD: standard deviation.

There was no significant difference between groups R and RD with respect to sedation measured with the Ramsay Scale immediately after shifting at the first, fourth, eighth, 12th, and 24th hours. A significant difference ('p' value<0.05) in Ramsay's sedation score was observed between groups R and RD at the 18th post-operative hour, possibly due to the requirement of a higher dose of morphine in group R. There was a significant difference in the Short Assessment of Patient Satisfaction Score (SAPS) with respect to the quality of analgesia between groups R and RD (Figure 4). The mean of patient satisfaction score in group R was 4.22 ± 0.93, whereas in group RD, it was 4.70 ± 0.50 (p=0.002), indicating better patient satisfaction in group RD.

## Discussion

Women undergoing cesarean delivery should have access to high-quality pain relief that is safe and effective [9]. As a part of multimodal analgesia, neuraxial narcotics are the most used modality for post-operative analgesia in patients undergoing cesarean sections. Intrathecal morphine is considered the gold standard for post-operative analgesia after cesarean delivery [10,11]. However, it is associated with complications like pruritus, nausea/vomiting, sedation, and respiratory depression [12]. TAP block is the most recent addition to the various techniques that use local anesthetic drugs to block the peripheral afferent nerves. Various drugs and adjuvants have been used in different studies to increase the duration and quality of analgesia in peripheral nerve blocks.

In our study, we found that the addition of dexmedetomidine to ropivacaine decreased the total dose of rescue analgesic (morphine) needed, increased the time to request for rescue analgesia, and prolonged the duration of analgesia as compared to patients who received only ropivacaine for the TAP block. A significant difference was observed between the two groups with respect to VAS-C and VAS-R at the 12th and 18th hour post-operatively (‘p’ value<0.05). It indicates that the addition of 1 microgram/kg dexmedetomidine to ropivacaine can prolong the post-operative analgesic effect of the TAP block up to 18 hours. Also, patients who received only ropivacaine needed rescue analgesia earlier (a mean time to rescue analgesia 13.12 ± 3.53 hours) as compared to those who received TAP block with dexmedetomidine and ropivacaine (mean time to rescue analgesia 18.21 ± 3.14 hours).

In 2014, Fritsch et al. conducted a study on 62 patients undergoing elective shoulder surgery under general anesthesia with an interscalene block. The median duration of the nerve block was 18 hours in patients who received dexmedetomidine with ropivacaine as compared to 14 hours in patients who received only ropivacaine [13]. In 2012, Swami et al. found similar results in their study conducted over sixty ASA I and II patients scheduled for elective upper limb surgeries under supraclavicular brachial plexus block. Duration of analgesia (time to requirement of rescue analgesia) in patients who received dexmedetomidine 1 μg/kg with bupivacaine group was 456 ± 97 min as compared to 289 ± 62 min in patients who received clonidine with bupivacaine [14]. In 2013, Lin et al. found similar results in a study on forty American Society of Anesthesiologists (ASA) Class I or II adult patients who underwent thyroid surgery and received cervical plexus block. They concluded that the addition of 1 μg/kg dexmedetomidine to ropivacaine for cervical plexus block extended the duration of analgesia [15]. In 2021, Singla et al. [16] compared the analgesic efficacy of dexamethasone versus dexmedetomidine as an adjuvant to ropivacaine in ultrasound-guided transversus abdominis plane block for post-operative pain relief in cesarean section and concluded that addition of dexmedetomidine to ropivacaine in TAP block significantly reduces initial post-operative pain and prolongs the time to first rescue analgesic with minimal adverse effects, as compared with dexamethasone in parturients undergoing lower segment cesarean section.

Dexmedetomidine is a highly selective central alpha-2 adrenergic agonist [16]. It has profound sedative, anxiolytic, and analgesic properties. It reduces the inflammation and prolongs the duration of nerve block through vasoconstriction and inhibiting hyperpolarisation-activated cationic current. Dexmedetomidine acts by blocking the conduction of nerve signals through C and A delta fibers and may stimulate the release of encephalin-like substances at peripheral sites. In this way, dexmedetomidine potentiates the local anesthetic effects and prolongs their analgesic duration [16]. Literature shows that dexmedetomidine can prolong the blockade of the peripheral nerve by 200 minutes in a dose of 1 μg/kg [17]. In a systematic review and meta-analysis by Abdallah and Brull [18], it has been shown that dexmedetomidine can further extend the action of long-acting local anesthetics used in spinal blocks. They have also mentioned that in the peripheral application category, dexmedetomidine even sometimes exceeds the effect of clonidine. Different doses of dexmedetomidine were used in these studies. The meta-analysis showed the presence of reversible bradycardia in less than 10 percent of cases. In our study, the addition of dexmedetomidine to ropivacaine as an adjuvant to ropivacaine for TAP block in patients undergoing cesarean section significantly increased the duration of post-operative analgesia.

Our study did have some limitations. There was no methodology to confirm the effectiveness or failure of the TAP block. Also, we could not establish if the effect of dexmedetomidine was due to its systemic absorption or by a perineural effect. Further studies comparing the effect of dexmedetomidine use in a TAP block versus an intravenous infusion would establish this. Lastly, there was no mechanism to know the exact duration of the sensory loss as the TAP block was administered during the period of anesthesia caused by the subarachnoid block.

## Conclusions

TAP block, as a constituent of multimodal analgesia, is a proven method to provide post-operative analgesia in patients undergoing cesarean section. Dexmedetomidine, when used as an adjuvant to ropivacaine in TAP block, significantly increased the duration of post-operative analgesia and was associated with better patient satisfaction. However, further studies may be required to establish the exact mechanism through which dexmedetomidine acts in prolonging the duration of analgesia.
